# The Effectiveness of Pemetrexed Monotherapy Depending on Polymorphisms in *TS* and *MTHFR* Genes as Well as Clinical Factors in Advanced NSCLC Patients

**DOI:** 10.1007/s12253-015-9966-z

**Published:** 2015-08-16

**Authors:** Tomasz Kucharczyk, Paweł Krawczyk, Tomasz Powrózek, Dariusz M. Kowalski, Rodryg Ramlau, Ewa Kalinka-Warzocha, Magdalena Knetki-Wróblewska, Kinga Winiarczyk, Maciej Krzakowski, Janusz Milanowski

**Affiliations:** Department of Pneumonology, Oncology and Allergology, Medical University of Lublin, Jaczewskiego 8, 20-954 Lublin, Poland; Postgraduate School of Molecular Medicine, Warsaw Medical University, Żwirki i Wigury 61, 02-091 Warszawa, Poland; Department of Lung and Chest Cancer, Oncology Centre-Institute, M. Sklodowska-Curie in Warsaw, W. K. Roentgena 5, 02-781 Warszawa, Poland; Greater Poland Center of Pulmonology and Thoracic Surgery of Eugenia and Janusz Zeyland, Poznań, Poland; Department of Clinical Oncology, Chair of Cardio-Thoracic Surgery, University of Medical Sciences, Szamarzewskiego 82/84, 60-569 Poznań, Poland; Regional Centre of Oncology in Łódź, Ignacego Paderewskiego 4, 90-993 Łódź, Poland; Institute of Agricultural Medicine of Lublin, Kazimierza Jaczewskiego 2, 20-950 Lublin, Poland

**Keywords:** Non-small cell lung cancer, Pemetrexed monotherapy, *TS*, *MTHFR*, Polymorphism

## Abstract

In NSCLC, second-line chemotherapy using pemetrexed or docetaxel has limited efficacy and should be dedicated to selected groups of patients. Pemetrexed is an antifolate compound with the ability to inhibit enzymes (TS, DHFR and GARFT) involved in pyrimidine and purine synthesis. The objective of this study was to evaluate the association between polymorphisms of *TS* and *MHFR* genes and clinical outcomes in NSCLC patients treated with pemetrexed monotherapy. DNA was isolated from peripheral blood of 72 non-squamous NSCLC patients treated with pemetrexed. Using PCR and RFLP methods, the variable number of tandem repeats (VNTR), the G > C SNP in these repeats and insertion/deletion polymorphism of *TS* gene as well as 677C > T SNP in *MTHFR* gene were analyzed and correlated with disease control rate, progression-free survival and overall survival (OS) of NSCLC patients. Carriers of 2R/3R(G), 3R(C)/3R(G), 3R(G)/3R(G) genotypes showed significantly more frequent early progression than carriers of 2R/2R, 2R/3R(C), 3R(C)/3R(C) genotypes of *TS* gene (*p* < 0.05). Among carriers of triple 28 bp tandem repeats (3R) in *TS* gene and C/C genotype of *MTHFR* gene a significantly shorter OS was observed (HR = 3.07; *p* = 0.003). In multivariate analysis, significantly higher risk of death was observed in carriers of both 3R/3R genotype in *TS* and C/C genotype in 677C > T SNP in *MTHFR* (HR = 3.85; *p* < 0.005) as well as in patients with short duration of response to first-line chemotherapy (HR = 2.09; *p* < 0.005). Results of our study suggested that genetic factors may have a high predictive and prognostic value (even greater than clinical factors) for patients treated with pemetrexed monotherapy.

## Introduction

Lung cancer is the most prevalent cause of death due to malignancies worldwide. There is very low percentage of early diagnosed NSCLC patients which results in only 15 % chance of surgical resection [1]. Systemic therapy (chemo- and radiotherapy) is considered to be the main form of lung cancer treatment. Unfortunately, first-line treatment is of low effectiveness and progression is observed in the vast majority of patients.

Pemetrexed is one of the novel 3rd generation drugs with the least side effects reported. In the first-line study, cisplatin with pemetrexed proved to be more effective in non-squamous histology of NSCLC and as a result it was registered in only such histology of lung cancer [2]. Second-line treatment with pemetrexed monotherapy is possible in non-squamous patients with good performance status who progressed after non-pemetrexed first-line chemotherapy, which was proven by a comparative study with docetaxel [3].

Pemetrexed, a multitarget antifolate, is effective in non-squamous NSCLC and malignant pleural mesothelioma, with the main focus on inhibition of enzymes involved in pyrimidine and purine synthesis such as thymidylate synthase (TS), dihydrofolate reductase (DHFR) and glycinamide ribonucleotide formyltransferase (GARFT). TS catalyzes transformation of dUMP into dTMP. Decreased levels of dTMP results in inhibition of DNA repair and synthesis, thus cell death [2–4].

Studies have shown that expression levels of pemetrexed target enzymes may alter the effectiveness of the drug [5, 6, 7]. Squamous cell carcinoma was shown to have higher expression of TS than adenocarcinoma, thus being more resistant to pemetrexed treatment [8, 9].

Free circulating folates may regulate pemetrexed targets’ activity. Increased level of methyltetrahydrofolate (5-methylTHF) is a result of changes in the activity of 5,10-methylenetetrahydrofolate reductase (MTHFR), which results in higher TS activity and thus reduces pemetrexed efficacy [10].

There are three well known polymorphic changes that affect TS mRNA level and TS protein expression: various number of 28-base pair tandem repeats (VNTR) located in the 5′ end untranslated region of *TS* gene; a single nucleotide polymorphism (SNP) G > C in the second repeat of the 28-bp tandem repeats; and a 6-bp deletion at the 3’ end of the *TS* gene (1494del6) [11–13]. Polymorphisms in *TS* gene are known to alter effectiveness of 5-fluorouracil (5-FU) in colorectal cancer [14]. Moreover, *MTHFR* gene polymorphism 677C > T is causing lower expression of MTHFR and decreased levels of 5-methylTHF in colon and breast cancer cell lines [15].

This retrospective, non-randomized, multicenter study was carried out in order to assess the usefulness of *TS* and *MTHFR* gene polymorphisms as predictive markers in NSCLC patients treated with pemetrexed monotherapy.

## Materials and Methods

The whole studied group consisted of 72 NSCLC patients (46 male, 26 female; median age 61) with non-squamous histology, who were treated with pemetrexed monotherapy. Clinical data was collected from all patients. 500 mg/m^2^ of pemetrexed was administered as an intravenous infusion on day 1 of each 21 day cycle. In order to reduce toxicity, patients received folic acid and B12 vitamin prior to treatment.

The response to chemotherapy was assessed according to RECIST criteria. The observation period was from 2008 until February 2014, when progression was observed in 60 patients of whom 45 died.

The material for the study were blood samples collected in four oncology centres in Poland. All genetic testing was performed in clinical laboratory in Pneumonology, Oncology and Allergology Department in Lublin. Venous blood samples of 5 ml have been collected to EDTA-covered tubes. DNA was extracted using QIAamp DNA Mini Kit (Qiagen, Germany).

The study was approved by the Ethics Committee of the Medical University in Lublin (No. KE-0254/219/2010).

### Genotyping of *TS* and *MTHFR*

All studied polymorphism were analysed by polymerase chain reaction (PCR). Primers used in *TS* gene VNTR polymorphism analysis were previously described by Iacopetta et al. [16]. The analysis of G > C SNP in tandem repeats of TS gene was carried out using allele-specific PCR (ASP-PCR) with forward primers designed specifically for G or C nucleotide (5’CGTCCCGCCGCGCCACTTG 3’ and 5’CGTCCCGCCGCGCCACTTC 3’, respectively). The reverse primer was the same one used in the VNTR analysis, described by Iacopetta et al. The ASP-PCR was carried out in two separate PCR tubes, one for G-ending primer and one for C-ending primer. The 1494del6 deletion on the 3’ end of *TS* gene and *MTHFR* 677C > T SNP were detected by restriction fragment length polymorphism (RFLP) technique. Primers used for the detection of 1494del6 were previously described by Dotor et al. [17], while the *MTHFR* 677C > T SNP primers were previously described by Frosst et al. [18]. For the RFLP reaction Fermentas FastDigest *DraI* (Thermo Scientific, USA) for *TS* 1494del6 polymorphism and Fermentas FastDigest *HinfI* (Thermo Scientific, USA) for *MTHFR* 677C > T SNP restriction enzymes were used. The digestion process was carried out under the following conditions: 10 min. incubtion in 37° followed by 5 min. digestion in 65°.

Every polymorphism analysis was carried out in the same reaction mixture of 20 μl, with only PCR primers being changed specifically for the polymorphism. The mixture contained: 100 ng of sample DNA, 1 μM of each primer, 0.2 mM of each dNTP, 2.4 mM of MgCl_2_ and 1 U Taq polymerase with 1 × Reaction Buffer (Thermo Scientific, USA). All reactions were carried out in a Biometra TPersonal thermocycler (Biometra, Germany).

Products of PCR were visualised on 2 % agarose gel with ethidium bromide. *TS* VNTR polymorphism PCR products differed according to the number of 28 bp repeats. In Caucasian population the most often described genotypes consist of two or three repeats: 2R/2R and 3R/3R homozygotes, and 2R/3R heterozygotes. 2R/2R homozygotes produce a 116 bp band on the gel, 3R/3R homozygotes produce a 144 bp band, and heterozygotes show both bands - 116 and 144 bp. The ASP-PCR for the G > C SNP in *TS* gene shows two band patterns: the common G nucleotide presented three bands: 124 and 96 bp specific for G-ending primer and 68 bp band specific for C-ending primer, the rare C nucleotide presented three bands of 124 bp for G-ending primer and two bands of 96 and 68 bp for C-ending primer. The reaction products of G > C SNP are shown in two separate lanes, one for G-ending primer and one for C-ending primer.

The 6 bp deletion at the 3’ end of *TS* gene shows two band types: 142 bp for −6/−6 genotype and 148 bp for +6/+6 bp. The PCR products digestion with *DraI* restriction enzyme is possible only when the insertion genotype is present, so it creates two smaller bands of 88 and 60 bp. The heterozygous genotype +6/−6 presents four bands of 148, 142, 88 and 60 bp. The products of PCR for 677C > T SNP in *MTHFR* gene present following patterns: 198 bp band for C/C genotype and 175 and 23 bp band for T/T genotype when digested with *HinfI* restriction enzyme. The C/T heterozygote is present on the gel as three band of 198, 175 and 23 bp. The smallest band of 23 bp is not visible on the gel as it is migrating on the gel with reaction primers.

### Statistical Analysis

Yates’ chi-square test was used to assess differences between categorical variables and deviation from the Hardy-Weinberg equilibrium. Groups of patients with different clinical and molecular factors were compared using Kaplan-Meier method, in order to determine the probability of progression-free survival (PFS) and overall survival (OS). To determine the influence of the studied factors on PFS and OS of patients treated with pemetrexed monotherapy, the Cox regression model with step-by-step selection was used.

## Results

The studied group consisted of NSCLC patients treated with pemetrexed monotherapy in second- or third-line and first-line patients who were not eligible for cisplatin. In this group there were 24 carriers of double 28 bp tandem repeats (33.3 %), 17 triple repeats (23.6 %), the rest (43.1 %) were heterozygous. Among the carriers of triple repeats in one or both alleles, the G nucleotide in the second repeat of 28 bp appeared in 25 patients (34.7 %). The presence of 1494del6 deletion was observed in 8 patients (11.1 %), there were 28 carriers of one allele with deletion (38.95 %) and the insertion was present in 50 % of patients. Allele T in 677C > T SNP in *MTHFR* gene was present in 40 patients (55.6 %) among which there were 11 T/T homozygous patients (15.3 % of the whole studied group).

66.7 % of the studied group showed disease control during pemetrexed monotherapy, of whom only 7 reached partial response (PR) (9.7 %). In 24 patients (33.3 %) disease progression was observed during first two months after pemetrexed monotherapy. Median progression-free survival reached 4.5 months, while median overall survival reached 9 months.

Surprisingly, among all the analyzed clinical factors, none has significantly influenced the risk of early disease progression or progression-free survival. Slightly prolonged overall survival (HR = 0.58, *p* = 0.076) was observed among patients with PFS longer than 6 months after first-line non-pemetrexed chemotherapy (Table 1).

**Table 1:** Correlation between clinical factors and risk of early progression, duration of PFS and OS in non-squamous NSCLC patients treated with pemetrexed monotherapy

Factor	No.	PD, n (%)	SD, PR, n (%)	pχ^2^	Median PFS (months)	pχ^2^	HR [95%CI]	Median OS (months)	p χ^2^	HR [95%CI]
**Whole group**	72	24 (33.3)	48 (66.7)	–	4.5	–	–	9	–	–
**Age (years)**										
≤61	32	13 (41)	19 (59)	0.3562 0.851	4.5	0.378 0.776	1.2421 [0.7288–2.1171]	12	0.455 0.556	1.2391 [0.6874–2.2338]
>61	40	11 (27.5)	29 (72.5)		5			11		
**Gender**										
Male	46	17 (37)	29 (63)	0.543 0.369	4	0.933 0.007	0.9786 [0.5722–1.6737]	10.5	0.870 0.026	1.0490 [0.5760–1.9103]
Female	26	7 (27)	19 (73)		6			12		
**Smoking status**										
Smoker	63	21 (33.3)	42 (66.7)	0.7503 0.143	4	0.481 0.494	0.7779 [0.3423–1.7679]	10.5	0.652 0.202	1.1983 [0.5353–2.6828]
Non-smoker	9	3 (33.3)	6 (66.7)		5			13.5		
**Performance status**										
PS = 0	19	9 (47.3)	10 (52.7)	0.2191 1.51	4	0.993 0.0001	1.0023 [0.5684–1.7674]	12.5	0.247 1.337	1.4321 [0.7737–2.6509]
PS = 1	53	15 (28.3)	38 (71.7)		5			10.5		
**Weight loss**										
>5 %	22	8 (36.4)	14 (63.6)	0.9281 0.008	4	0.680 0.169	1.1204 [0.6191–2.0274]	12	0.763 0.090	1.1035 [0.5609–2.1710]
≤5 %	50	16 (32)	34 (68)		5.25			11		
**Anemia**										
Yes	20	9 (45)	11 (55)	0.3061 1.047	3.25	0.146 2.111	1.4729 [0.8010–2.7084]	9.5	0.215 1.538	1.4512 [0.7303–2.8840]
No	52	15 (29)	37 (71)		5.5			12.5		
**Disease stage**										
Inoperable	28	9 (32.1)	19 (67.9)	0.9333 0.007	5	0.740 0.109	0.9206 [0.5398–1.5701]	10	0.584 0.298	0.8540 [0.4684–1.5571]
Advanced (IV)	44	15 (34.1)	29 (65.9)		5			11		
**Prior surgical treatment**										
Yes	23	8 (34.8)	15 (65.2)	0.9287 0.008	5	0.489 0.478	0.8258 [0.4735–1.4400]	13	0.532 0.389	0.8217 [0.4416–1.5289]
No	49	16 (32.7)	33 (67.3)		5			10.5		
**Prior radiotherapy**										
Yes	36	13 (36.1)	23 (63.9)	0.8034 0.062	4	0.285 1.141	1.3068 [0.7771–2.1976]	10.5	0.679 0.170	1.1280 [0.6283–2.0249]
No	36	11 (30.6)	25 (69.4)		5			11		
**Response to first-line no-pemtrexed treatment**										
PD	8	4 (50)	4(50)	0.8336 0.364	5	0.8125 1.4153	–	8.75	0.8485 0.3285	
SD	29	9 (31)	20 (69)		5			13		
PR	26	9 (34.6)	17 (65.4)		5.25			10		
**Cycles number of first-line no-pemetrexed therapy**										
<4 cycles	19	6 (31.6)	13 (68.4)	0.9383 0.006	6	0.3935 0.7282	1.2585 [0.7117–2.2254]	11	0.864 0.02934	1.0572 [0.5633–2.0201]
≥4 cycles	44	16 (36.4)	28 (63.6)		5			10.5		
**Duration of PFS after first-line no-pemetrexed therapy**										
<6 months	19	11 (57.9)	8 (42.1)	0.4436 0587	2	0.1336 2.2502	0.6597 [0.3447–1.2626]	7	0.0762 3.1442	0.5796 [0.2865–1.1726]
≥6 months	44	12 (27.3)	32 (72.7)		5.5			12		

**Fig. 1:**
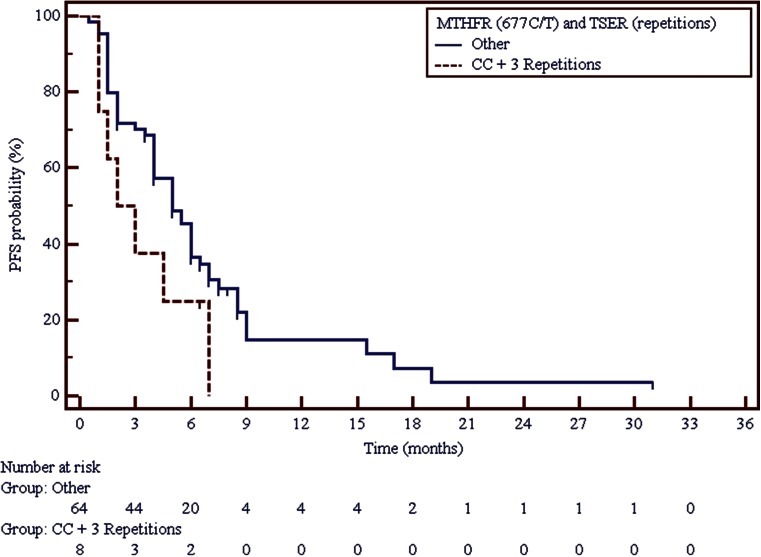
Impact of *TS* VNTR polymorphism and a 677C > T SNP in *MTHFR* gene on progression free survival in non-squamous NSCLC patients treated with pemetrexed monotherapy

It was observed that among carriers of triple 28 bp tandem repeats with G allele in the second tandem repeat, disease progression was significantly more frequent (*p* = 0.0287) than among carriers of other genotype combinations. PFS and OS were shorter in carriers of such genotype, however these were not statistically significant differences (Table 2). Only among carriers of both triple tandem repeats in *TS* gene and C/C genotype in *MTHFR* 677C > T polymorphism, a double shortening of PFS may be observed in comparison to carriers of other genotype combinations. However, this difference was not statistically significant either (Fig. 1, Table 2).

**Table 2:** Correlation between molecular factors and risk of early progression, duration of PFS and OS in non-squamous NSCLC patients treated with pemetrexed monotherapy

Factor	No.	PD, n (%)	SD, PR, n (%)	P χ^2^	Median PFS (months)	p χ^2^	HR [95%CI]	Median OS (months)	p χ^2^	HR [95%CI]
***TS*** **gene polymorphism (repetitions)**										
2R/2R	24	6 (25)	18 (75)	0.7722 0.517	6	0.572 1.114	−	12	0.263 2.668	−
2R/3R	31	10 (32.3)	21 (67.7)		5			12		
3R/3R	17	8 (47.1)	9 (52.9)		4			9		
***TS*** **gene polymorphism (repetitions)/SNP in the second repeat of** ***TS*** **promoter region**										
2R/2R	24	5 (20.8)	19 (79.2)	0.1119 7.495	6	0.447 3.702	−	12	0.614 2.668	−
2R/3R(G)	8	3 (37.5)	5 (62.5)		4			12		
2R/3R(C)	23	6 (26.1)	17 (73.9)		5.5			11		
3R(G)/3R(C)	13	9 (69.2)	4 (30.8)		2			9		
3R(G)/3R(G)	4	1 (25)	3 (75)		6.75			12		
2R/2R + 2R/3R(C)	47	11 (23.4)	36 (76.6)	0.0287 4.787	5	0.802 0.063	0.9389 [0.5586–1.5782]	12	0.643 0.215	0.8717 [0.4834–1.5720]
2R/3R(G) + 3R(C)/3R(G) +3R(G)/3R(G)	25	13 (52)	12 (48)		4,5			10,5		
***TS*** **1494del6 genotypes**										
−/− 6 bp	8	2 (25)	6 (75)	0.9748 0.051	4.25	0.409 1.787	−	8.5	0.424 1.713	−
+/− 6 bp	28	10 (35.7)	18 (64.3)		4			10		
+/+ 6 bp	36	12 (33.3)	24 (66.7)		6			13.5		
+/+ 6 pz	36	12 (33.3)	24 (66.7)	0.818 0.053	6	0.139 2.194	1.4225 [0.8381–2.4143]	13.5	0.179 1.806	1.4761 [0.8212–2.6532]
−6/− 6 bp and +6/− 6 bp	36	13 (36.1)	23 (63.9)		4			10		
***MTHFR*** **(SNP 677C > T) genotypes**										
C/C	32	10 (31.25)	22 (68.75)	0.5822 1.082	5.5	0.391 1.877	−	11	0.884 0.247	−
C/T	29	12 (41.4)	17 (58.6)		4			13.5		
T/T	11	2 (18.2)	9 (81.8)		5			17.5		
C/C	32	10 (31.25)	22 (68.75)	0.8875 0.9092	5.5	0.220 1.501	1.3595 [0.8088–2.2849]	11	0.620 0.244	0.8668 [0.4805–1.5636]
C/T + T/T	40	12 (40)	28 (60)		4			13.5		
T/T	11	2 (18.2)	9 (81.8)	0.4176 0.657	5	0.865 0.028	1.0595 [0.5271–2.1297]	17.5	0.749 0.102	1.1362 [0.5249–2.4597]
C/T + C/C	61	22 (36.1)	39 (63.9)		5			11		
***MTHFR*** **(SNP 677C > T) genotypes and** ***TS*** **gene polymorphism (repetitions)**										
C/C + 3R	8	4 (50)	4 (50)	0.5076 0.439	2.5	0.081 3.036	1.9150 [0.6834–5.3657]	6.75	0.003 8.763	3.0698 [0.8670–10.8692]
Other	64	20 (31.2)	44 (68.8)		5			12

**Fig. 2:**
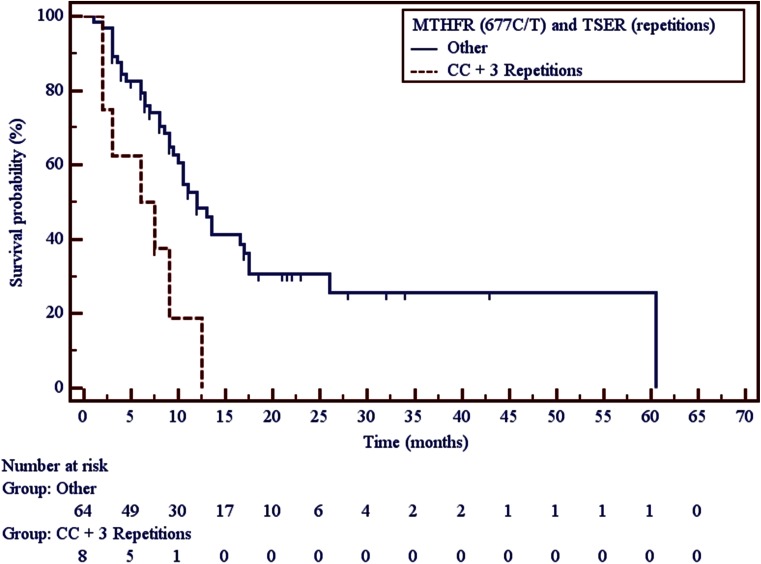
Impact of *TS* VNTR polymorphism and a 677C > T SNP in *MTHFR* gene on overall survival in non-squamous NSCLC patients treated with pemetrexed monotherapy

Carriers of both triple 28 bp tandem repeats in both alleles of *TS* gene and C/C genotype in 677C > T SNP in *MTHFR* gene had significantly shorter overall survival in comparison to carriers of other genotype combinations (6.75 vs. 12 months, *p* = 0.0031) (Fig. 2, Table 2).

Using Cox logistic regression we were unable to create a model evaluating the power of the impact of the studied clinical and molecular factors on the risk of early progression due to the lack of significance of each determinant. The model created for the risk of early death has reached the significance threshold, however the only variable, which significantly influenced this risk, was the presence of both triple 28 bp tandem repeats in *TS* gene and C/C genotype in 677C > T SNP in *MTHFR* gene as well as short duration of PFS after first-line non-pemetrexed chemotherapy (Table 3).

**Table 3:** Multivariate Cox logistic regression model of factors affecting the overall survival of non-squamous NSCLC patients treated with pemetrexed monotherapy

	β	p	HR [95%CI]
**C/C genotype of** ***MTHFR*** **SNP 677C > T + 3R/3R genotype of TS gene**	1.3469	0.0042	3.8456 [1.5364–9.6256]
**Short duration of PFS after first-line no-pemetrexed chemotherapy (<6 months)**	0.7385	0.00299	2.0928 [1.0782–4.0622]

**Overall fit model:** *p* = 0.00082, χ^2^ **=** 9.606

## Discussion

In second-line therapy, pemetrexed is administered as monotherapy, and this form was registered based on a large-scale, multicenter, randomized, phase III clinical trial performed on 571 NSCLC patients. The study compared the effectiveness of second-line pemetrexed or docetaxel monotherapy and it did not show differences in efficacy of the drugs, however significantly less toxicity was observed after pemetrexed treatment. In pemetrexed arm objective response and stable disease was observed in 71.2 % of the studied group, PFS reached 2.9 months and OS – 8.3 months. The study also revealed that the basic clinical factors such as good performance status (PS = 0), locally advanced disease and time since last chemotherapy over 3 months are positively influencing overall survival in NSCLC patients treated with pemetrexed monotherapy [3].

Pemetrexed monotherapy was compared with a pemetrexed/carboplatin regime, in second-line of NSCLC treatment, in GOIRC 02–2006 clinical trial. PFS in the pemetrexed arm reached 3.6 months and OS – 8.8 months, which were similar to the result of the Hanna et al. trial. Two-drug regime showed slightly higher effectiveness but for a price of higher toxicity [19].

Maintenance therapy using pemetrexed is possible in selected non-squamous NSCLC patients. The PARAMOUNT clinical trial showed that pemetrexed monotherapy after successful first-line pemetrexed/platinum therapy, extends progression-free survival to 4.1 months and overall survival to 13.9 months [20].

In our study, we reached results similar to the ones obtained by Hanna et al. Median PFS in patients treated with pemetrexed monotherapy was 4.5 months and OS – 9 months. Such results were reached even though only 18 % of our studied group received further treatment lines. Only slight but not significant differences in OS are visible between patients with different PS (12.5 *vs.* 10.5 months for PS = 0 and PS = 1, respectively), in patients with or without anemia (9.5 *vs.* 12.5 months) and in patients with long and short response to first-line non-pemetrexed chemotherapy (12 *vs.*7 months).

Clinical factors are important in proper qualification to chemotherapy but it is not always possible to choose the best option based only on them, thus molecular factors are being studied more and more intensively in order to find relevant ones, also in pemetrexed treatment.

The most often studied molecular factor is the expression of thymidylate synthase. A study on 110 NSCLC patients treated with pemetrexed in third- or fourth-line did not show any significant influence of TS expression on PFS or OS. However it is worth mentioning that the researchers had only limited access to cancer tissue (only 13 out of 55 samples were eligible for immunohistochemical analysis) [21].

Analysis of pemetrexed target genes (*TS*, *DHFR*, *GARFT* and *MTHFR*) was carried out on 90 adenocarcinoma Asian patients treated with pemetrexed monotherapy in third- or further lines. No relationship was found between the polymorphisms and response rates, but it is interesting that carriers of “high expression” genotypes in *TS* gene (2R/3R(G), 3R(C)/3R(G), 3R(G)/3R(G)) reached longer PFS (5.2 vs*.* 3.7 months) and OS (31.8 vs*.* 18.5 months) than carriers of “low expression” genotypes (2R/2R, 2R/3R(C), 3R(C)/3R(C)), which is in contradiction to the results from the studies of the same polymorphism but in first-line pemetrexed treatment [22].

Smit et al. carried out a comparison of pemetrexed monotherapy with pemetrexed/carboplatin regime in second-line treatment of NSCLC patients. As a part of the study a pharmacogenetic analysis was carried out. There was no connection between “low/high expression” genotypes of *TS* gene and treatment results, but a significant correlation was observed in carriers of T/T genotype of *MTHFR* 677C > T polymorphism, where PFS was longer for such patients when compared to carriers of other genotypes (7.9 vs*.* 2.9 months; *p* = 0.003) [23].

The analysis of two clinical trials: GOIRC 02–2006 (19) and NVALT-7 (23), for polymorphisms connected with pemetrexed effectiveness, showed that T/T genotype of *MTHFR* 677C > T polymorphism is correlated with longer PFS (5.4 vs*.* 3.4 months; *p* = 0.012) and OS (16.4 vs*.* 8.5 months; *p* = 0.026) when compared to other genotypes (C/C and C/T) [24].

In our study carriers of “high expression” genotypes in *TS* gene showed significantly more frequent early progression than carriers of “low expression” genotypes. Small (but not statistically significant) differences in OS were observed among carriers of different genotypes of *MTHFR* gene 677C > T polymorphism (17.5 vs*.* 11 months for carriers of T/T and C/C + C/T, respectively). Among carriers of 3R/3R genotype in *TS* gene and C/C genotype of *MTHFR* 677C > T polymorphism a significantly shorter OS was observed (6.75 vs*.* 12 months for carriers of 3R/3R + C/C and carriers of other genotype combinations respectively; *p* = 0.0031). Moreover, in multivariate analysis, significantly higher risk of death was observed in carriers of both 3R/3R genotype in *TS* gene and C/C genotype in 677C > T SNP in *MTHFR* gene as well as in patients with short PFS after first-line non-pemetrexed chemotherapy. These results are in line with other studies, where the presence of 3R/3R genotype in *TS* gene and C/C genotype in *MTHFR* gene where considered negative predictive factor in pemetrexed therapy.

Molecular factors become more meaningful when considering a relevant treatment option for cancer patients. Studies carried out on first-line pemetrexed/platinum treated patients seem to confirm usefulness of some factors such as thymidylate synthase expression, but in pemetrexed monotherapy group there is still very little relevant information. It is also worth mentioning that patients who receive second or further lines of chemotherapy usually have worse prognosis than those who are treated with first-line. Therefore, based on our study, it appears that the studied genetic factors may have high predictive and prognostic value (as high as clinical factors). Our results show that analysis of VNTR polymorphism in *TS* gene, an inside G > C SNP in VNTRs and *MTHFR* gene 677C > T SNP might help in selecting a group of non-squamous NSCLC patients who will benefit significantly from pemetrexed monotherapy, but in order to evaluate properly the usefulness of the chosen molecular factors more large-scale and prospective studies on more representative groups are needed.
